# The Black Locust Tree: Toxalbumin-Induced Tissue Necrosis of the Upper Extremity

**DOI:** 10.7759/cureus.11758

**Published:** 2020-11-28

**Authors:** Donald T Browne, Gabriela Aguilo-Seara, Anthony DeFranzo Jr.

**Affiliations:** 1 Plastic and Reconstructive Surgery, Wake Forest School of Medicine, Winston-Salem, USA

**Keywords:** black locust tree, necrotizing fasciitis, robin exposure, toxalbumin, forearm infection, streptococcus constellatus, eikenella corrodens

## Abstract

We present the case of a 48-year-old male who developed tissue necrosis and subsequent necrotizing fasciitis in his right upper extremity after sustaining a puncture injury from a large black locust tree (*Robinia pseudoacacia*) splinter. Blood and intraoperative wound cultures revealed *Streptococcus constellatus* and *Eikenella corrodens* infection. The treatment consisted of IV antibiotics, fasciotomy, and multiple debridements, which left a 30 x 5-cm defect, requiring negative pressure wound therapy with Integra Dermal Regeneration Template (Integra Lifesciences, Plainsboro Township, NJ) and, ultimately, split-thickness skin grafts. Although uncommon, plastic surgeons should be aware of tissue necrosis associated with robin toxalbumin in cases of black locust tree puncture wounds. Robin toxalbumin causes cellular death by inhibiting protein synthesis. In this patient, the toxalbumin from the black locust tree fragment led to extensive tissue necrosis, serving as the nidus for necrotizing fasciitis.

## Introduction

Necrotizing soft tissue infections of the forearm constitute a serious surgical emergency. These infections require early and aggressive surgical debridement, in addition to antibiotic therapy, to be adequately controlled [1]. The bacterial synergy in most soft tissue infections and poor diagnostic testing [2] can lead to deep fascial and varying degrees of muscle involvement [3]. In this case report, we present a case of extensive tissue necrosis with subsequent necrotizing soft tissue infection, secondary to black locust tree (*Robinia pseudoacacia*) exposure.

## Case presentation

**Figure 1:**
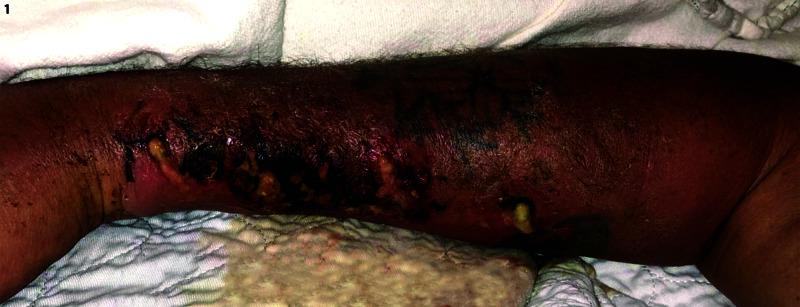
Patient’s right forearm on initial presentation with purulent drainage from the puncture site Note the distal spread of skin necrosis and infection. The orientation is the same for all figures, distal is left and proximal is right

A 48-year-old right-hand-dominant male presented to the emergency department at our institution with chief complaints of increasing pain, swelling, and foul-smelling drainage from his right upper extremity. He worked for a timber company and reported that 10 days earlier he had sustained a penetrating injury from a sharp wood fragment of the black locust tree. He had been felling a large black locust tree when a branch fell, and a wood fragment had gone on to penetrate his right forearm. The patient reported that four days after the exposure, the wood fragment had extruded when he squeezed his arm. The fragment was approximately 2-3 inches long. On presentation to the emergency room, the patient was febrile to 102.5 °F, had a heart rate of 101 beats per minute, and was normotensive, with a white blood count of 11.1. His exam demonstrated significant global edema of the right arm and hand. Associated erythema extended from the anti-cubital fossa through the entire forearm and to the level of the metacarpal phalangeal joints of the dorsal hand. A puncture wound was noted to be draining significant foul-smelling, purulent fluid. Skin breakdown and necrosis distal to the wound were also observed (Figure 1). Additionally, the exam revealed numbness and tingling in his index and long fingertips. His digits, wrist, and elbow were held in flexion. Active and passive flexion and extension of the fingers and wrist were limited by severe pain. The patient demonstrated symptoms of acute carpal tunnel secondary to hand swelling. Blood cultures were obtained. He was started on empiric IV antibiotics, including vancomycin, piperacillin/tazobactam, and clindamycin.

**Figure 2:**
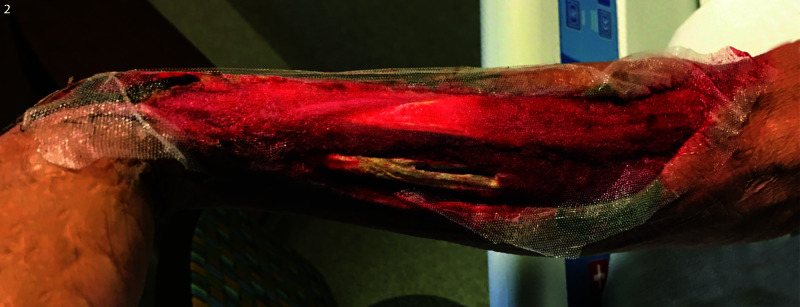
Postoperative image after the infection was surgically controlled and wound treated with negative pressure wound therapy This photo was taken on postoperative day 12

He was taken emergently for wound exploration and irrigation and debridement of the necrotic tissue. Excisional debridement of necrotic skin, subcutaneous tissue, fascia, and muscle; fasciotomies of the flexor and extensor compartments of the forearm; and carpal tunnel release were performed. There was extensive soft tissue necrosis in the patient’s forearm. The patient required debridement of the flexor digitorum superficialis at his initial operation. Of note, at the index operation, there were small fragments of tree bark and wood noted in the wound. Additionally, the patient’s cephalic vein was filled with puss, both distally and proximally to the puncture site. This segment of the vein was excised in addition to the nonviable skin, subcutaneous tissue, muscle, and fascia (Figure 2).

**Figure 3:**
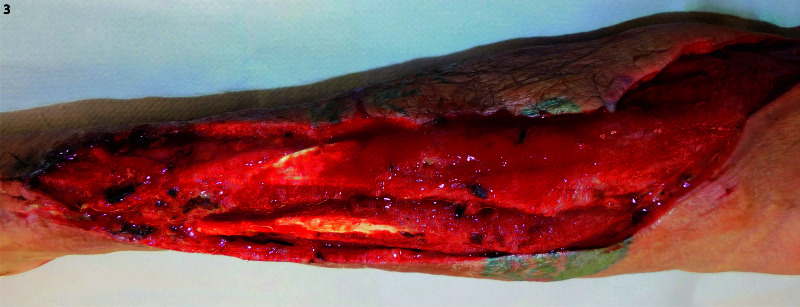
Radial view of right upper extremity wound prior to the placement of Integra Dermal Regeneration Template* *Integra Lifesciences, Plainsboro Township, NJ

**Figure 4:**
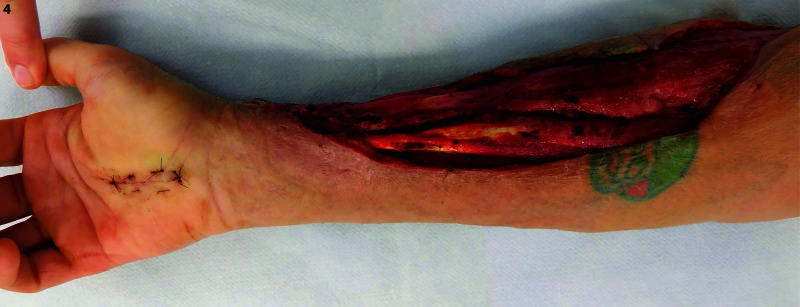
Anterior-posterior view of the right upper extremity wound prior to the placement of Integra Dermal Regeneration Template

Postoperatively, he was transferred to the surgical ICU. His blood cultures from the emergency department demonstrated *Streptococcus constellatus* bacteremia, and Infectious Disease service was consulted. Two intraoperative tissue cultures demonstrated *Streptococcus constellatus* and *Eikenella corrodens*. He was transitioned to targeted antibiotic therapy based on his culture results. His fever and white blood cell count improved; however, his forearm required two additional debridements, and negative pressure wound therapy was initiated following the third debridement, with two changes prior to takedown seven days later. In subsequent trips to the operating room, a more proximal purulent thrombophlebitis of the cephalic vein was again noted and debrided. Final forearm debridement resulted in a 30 x 5-cm defect, as seen in Figure 3 and Figure 4.

**Figure 5:**
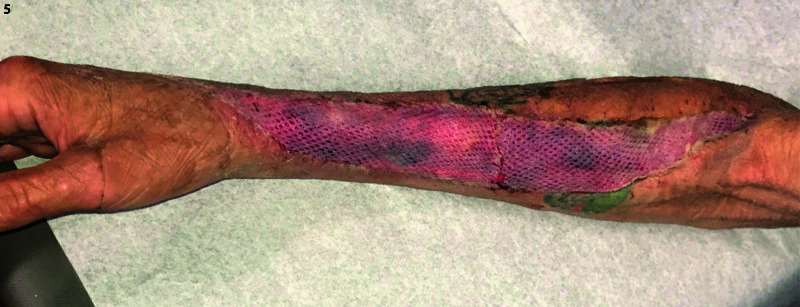
Postoperative view of the final reconstruction with Integra Dermal Regeneration Template and split-thickness skin grafting of the right upper extremity wound

Integra Dermal Regeneration Template and a wound vacuum-assisted closure (VAC) were placed on hospital day 11, as seen in Figure 5. The Integra was perforated with an 11-blade scalpel through the silicone sheet and the bi-layer matrix. The patient was discharged home 14 days after admission. Seven days after Integra and VAC therapy, he was brought back for placement of definitive split-thickness skin grafts as an outpatient procedure and an additional seven days of VAC therapy.

His skin grafts healed well with >95% take. However, he lost any meaningful function of his hand. At one year postoperatively, the patient reported that he still had very little function of his right hand with severe weakness. He is no longer able to make a fist and struggles to perform activities of daily living. His wrist flexion and extension are intact, and he is able to make a weak composite fist with limited active extension of his middle finger. However, he has difficulty lifting objects heavier than 5 pounds and cannot type. The patient has adapted to perform many activities with his left hand. He was originally uninsured and has declined to undergo hand therapy while he engages in a workers’ compensation lawsuit. His current Quick Disabilities of the Arm, Shoulder, and Hand (QuickDASH) [4] score is 77, indicating severe disability.

## Discussion

Toxalbumins are highly toxic, naturally occurring plant chemicals. The most common toxalbumin exposures include ricin from the castor bean, as well as robin and phasin from the black locust tree (*Robinia pseudoacacia*) [5]. The black locust tree, originally native to the Southeastern United States (US), is now an invasive species found all over the world. The entirety of the black locust possesses the toxalbumin robin, including the tree’s bark, wood, stems, seeds, and leaves. Robin, however, is mostly concentrated in the bark [6]. A 12-year review of poison center data by Kaland et al. demonstrates that the black locust accounts for the second most common toxalbumin exposure in the US [6]. The majority of toxalbumins exposures today involve ingestion [7]. However, 28.5% of all *Robinia pseudoacacia* exposures are dermal, and of the dermal exposures, 85.2% involve a puncture wound [6]. Minor effects of dermal toxalbumin exposure have included wounds, erythema, numbness, and edema. Among those with dermal exposure to toxalbumins parenterally, the effects have included edema, erythema, rash, tachycardia, fever, seizures, paralysis, and numbness [6]. Kaland et al. have also discussed one patient in their case series who had a dermal injection of the toxalbumin ricin, whose toxic properties are well documented. This particular patient developed pain and necrosis at injection sites requiring debridement, fasciotomies, and antibiotics, similar to our patient [6].

Toxalbumins work by a unique mechanism to inhibit protein synthesis and ultimately cause cell death. The toxin is a glycoprotein of two peptide chains, A and B respectively. The B chain is responsible for cell surface binding and endocytosis. The A chain inactivates ribosomes via glycosidase mechanism binding to 28S ribosome, impairing protein synthesis, and potentiating cellular death [5-8]. It is hypothesized that much of the toxicity is related to endothelial damage, causing increasing capillary permeability, edema, and protein leakage [9]. From a physiologic standpoint, clindamycin and other antibiotics that inhibit protein synthesis could be effective in inhibiting toxin production and controlling the inflammatory response that propagates the necrotizing soft tissue infection. However, there are no reports in the literature to support its use in toxalbumin exposure [10].

We hypothesize that the patient’s injury mechanism and delayed presentation led to his overall clinical picture. We believe that the cytotoxic protein introduced by the black locust splinter and residual bark fragments in the wound allowed robin toxalbumin to cause significant cellular damage. The resulting severe tissue necrosis served as the original nidus for secondary necrotizing fasciitis. The patient denied IV drug use as a possible mechanism of bacterial skin penetration, and urine drug screening confirmed this. Given the patient’s occupation and the puncture mechanism, it is logical to conclude the toxalbumin exposure was a major contributing factor to the cause of necrotizing fasciitis.

This patient has had a very poor outcome following surgery. His hand and upper extremity functions have been greatly reduced, as indicated by his QuickDASH [4] score of 77, suggesting severe disability and dysfunction. The patient has had limited follow-up after surgery. He initially declined hand therapy and was not interested in active therapy to improve his hand function. It is possible that surgical factors could have contributed to his poor function, such as adhesions from the use of Integra Dermal Matrix over the exposed tendon, although this is considered safe in the literature [11]. Additionally, studies of wartime combat injuries have shown that Integra Dermal Regeneration Template provides necessary coverage to vital structures and allows glide and motion of underlying muscle and tendinous structures [12]. It is most likely that the lack of follow-up and postoperative hand therapy has compounded the already devastating effect of toxalbumin and necrotizing soft tissue infection that led to this poor outcome.

As a case report, this study has a number of limitations. While they are excellent for sharing unusual or unexpected presentations or pathophysiology, case reports in general have many shortcomings due to their retrospective nature and large potential for overinterpretation. They cannot be used as a basis for generalizations, nor do they allow for the determination of cause-effect relationships, both of which hold true for this study.

## Conclusions

Surgeons evaluating wounds and infections should be aware of the danger that injuries caused by black locust trees can lead to. Black locust toxalbumin exposure is certainly not the first etiology that comes to mind when discussing necrotizing fasciitis. However, patients who present with a similarly deep, prolonged puncture injury from the black locust tree warrant further attention regarding the early signs and symptoms of progressing cell death and infection, and more aggressive initial management and debridement should be considered. The hand and upper extremity are some of the most common places that are injured, and surgeons need to be aware of the potential for progression to limb or even life-threatening injury.
